# Sesquiterpene Lactones Downregulate G2/M Cell Cycle Regulator Proteins and Affect the Invasive Potential of Human Soft Tissue Sarcoma Cells

**DOI:** 10.1371/journal.pone.0066300

**Published:** 2013-06-14

**Authors:** Birgit Lohberger, Beate Rinner, Nicole Stuendl, Heike Kaltenegger, Bibiane Steinecker-Frohnwieser, Eva Bernhart, Ehsan Bonyadi Rad, Annelie Martina Weinberg, Andreas Leithner, Rudolf Bauer, Nadine Kretschmer

**Affiliations:** 1 Department of Orthopedic Surgery, Medical University of Graz, Graz, Austria; 2 Center for Medical Research, Medical University of Graz, Graz, Austria; 3 Ludwig Boltzmann Institute for Rehabilitation Internal Diseases, Saalfelden, Austria; 4 Institute of Molecular Biology and Biochemistry, Medical University of Graz, Graz, Austria; 5 Cancer Biology Unit, Department of Dermatology, Medical University of Graz, Graz, Austria; 6 Department of Pediatric and Adolescence Surgery, Medical University of Graz, Graz, Austria; 7 Department of Pharmacognosy, Institute of Pharmaceutical Sciences, Karl-Franzens- University Graz, Graz, Austria; Univ of Bradford, United Kingdom

## Abstract

Soft tissue sarcomas (STS) represent a rare group of malignant tumors that frequently exhibit chemotherapeutic resistance and increased metastatic potential. Many studies have demonstrated the great potential of plant-derived agents in the treatment of various malignant entities. The present study investigates the effects of the sesquiterpene lactones costunolide and dehydrocostus lactone on cell cycle, MMP expression, and invasive potential of three human STS cell lines of various origins. Both compounds reduced cell proliferation in a time- and dose-dependent manner. Dehydrocostus lactone significantly inhibited cell proliferation, arrested the cells at the G2/M interface and caused a decrease in the expression of the cyclin-dependent kinase CDK2 and the cyclin-dependent kinase inhibitor p27^Kip1^. In addition, accumulation of cells at the G2/M phase transition interface resulted in a significant decrease in cdc2 (CDK1) together with cyclin B1. Costunolide had no effect on the cell cycle. Based on the fact that STS tend to form daughter cell nests and metastasize, the expression levels of matrix metalloproteinases (MMPs), which play a crucial role in extracellular matrix degradation and metastasis, were investigated by Luminex® technology and real-time RT-PCR. In the presence of costunolide, MMP-2 and -9 levels were significantly increased in SW-982 and TE-671 cells. Dehydrocostus lactone treatment significantly reduced MMP-2 and -9 expression in TE-671 cells, but increased MMP-9 level in SW-982 cells. In addition, the invasion potential was significantly reduced after treatment with both sesquiterpene lactones as investigated by the HTS FluoroBlock™ insert system.

## Introduction

*Saussurea lappa* Clarke (Asteraceae) is a plant traditionally used in different Asian medicine systems. Sesquiterpene lactones, including costunolide and dehydrocostus lactone, are major components of the roots and have been reported to exhibit a variety of biological activities [1]–[4]. It has also been reported that sesquiterpene lactones induced G2 arrest in human cancer cells, which may be correlated with the induction of apoptosis [5]–[8]. Our previous study has shown that dehydrocostus lactone inhibited cell proliferation and caused an enhanced caspase 3/7 activity, cleaved caspase-3, and cleaved PARP, indicating apoptosis induction in human sarcoma cell lines, and led to a G2/M phase arrest [9]. However, the molecular responsibility for the therapeutic activity and the cellular mechanisms underlying the action of sesquiterpene lactones in the induction of cell cycle arrest in sarcoma cells remains unknown. Cell proliferation is a tightly controlled process consisting of multiple checkpoints responsible for the regulation of abnormal cell cycle progression. Transitions between G1, S, and G2/M phases are regulated by biochemically coordinated actions of cyclins, cyclin-dependent kinases (CDKs), CDK inhibitors, all of which can in turn be modulated by diverse intracellular signals transduced from extracellular growth cues [10].

Matrix metalloproteinases (MMPs) are a family of zinc-dependent endopeptidases and major enzymes in extracellular matrix degradation. Pathologically, they are associated with arthritis, autoimmune diseases, fibrosis, heart failure and cancer [11], [12]. MMPs are assumed to regulate changes in the tumor microenvironment leading to tumor growth, progression, invasion, metastasis and angiogenesis. For this reason, they have become novel therapeutic targets for the treatment of cancer [13]–[15]. Particularly soft tissue sarcomas (STS) are a rare class of malignant tumors of various histologies with mostly aggressive characteristics both locally and in the formation of distant metastases. STS frequently exhibit chemotherapeutic resistance and an increased metastatic potential following unsuccessful cancer treatment. Since the efficacy of chemotherapeutic agents in STS is limited, there is an urgent need for the development and discovery of new lead substances [16], [17].

The aim of the present study was to investigate the cellular mechanisms underlying the observed cell cycle arrest, the effect of costunolide and dehydrocostus lactone on MMP expression and the invasive potential of three human STS cell lines of various origins.

## Materials and Methods

### Isolation of Costunolide and Dehydrocostus Lactone

Costunolide and dehydrocostus lactone were isolated from roots of *Saussurea lappa* Clarke as described previously [9]. In brief, freshly powdered roots were exhaustively extracted with petroleum ether by Soxhlet extraction and dried under reduced pressure. Costunolide and dehydrocostus lactone were isolated by means of preparative HPLC using a VDSpher 100 RP18 column (250×25 mm, 10 µm), a mobile phase consisting of A: water and B: acetonitrile, and the following gradient: 0–10 min: 84% B, 10–15 min: 84–100% B. Structure elucidation was done using a Varian Unitylnova 400 MHz (400 MHz for ^1^H and 100 MHz for ^13^C) Spectrometer at 25°C using TMS as the internal standard. Both were measured in pyridine-d_5_ (Sigma-Aldrich, MO, USA).

### Sample Preparation

Compounds were dissolved in DMSO and diluted with culture medium. The final DMSO concentration was max. 0.5%, which did not affect the behavior of the cells as observed by benchmark experiments. Vehicle-treated cells served as a control.

### Cell Culture

SW-872 (human liposarcoma), SW-982 (human synovial sarcoma) and TE-671 (human rhabdomyosarcoma) cell lines were obtained from CLS (Eppelheim, Germany) and cultured in Dulbecco’s-modified Eagle’s medium (DMEM-F12; GIBCO®, Invitrogen, Darmstadt, Germany), containing 5% fetal bovine serum (FBS; GIBCO®, Invitrogen), 1% L-glutamine (GIBCO®, Invitrogen), 100 units/ml Penicillin (GIBCO®, Invitrogen), 100 µg/ml Streptomycin (GIBCO®, Invitrogen) and 0.25 µg Amphotericin B (PAA Laboratory, Pasching, Austria). Cells were kept at 37°C in a humidified atmosphere of 5% CO_2_ and passaged by trypsination upon reaching confluence.

### Cell Proliferation

The xCELLigence DP device from Roche Diagnostics (Mannheim, Germany) can be used to quantitatively and dynamically monitor cell proliferation in real-time [18]. Respectively, 1×10^4^ cells were seeded in electronic microtiter plates (E-Plate™; Roche Diagnostic), treated with 3 and 10 µg/ml of each compound, and measured for 48 h with the xCELLigence system according to the instructions in the user’s manual. Application of a low-voltage (less than 20 mV) AC signal leads to the generation of an electric field that interacts with the ionic environment inside the wells of the E-Plates and is differentially modulated by the number of cells in the well, the morphology of the cells, and the strength of cell attachment. Cell density measurements were performed in triplicates with programmed signal detection every 20 min and normalized to the 6 h time point. Data acquisition and analysis was performed with the RTCA software (version 1.2, Roche Diagnostics).

### Cell Cycle Analysis by FACS Experiments

After incubation with the respective IC_50_ of both compounds for 48 h, cells were harvested by trypsinization. Ice cold ethanol (70%) was used to fix 5×10^5^ cells for 10 min at 4°C. After washing, the cell pellet was resuspended in propidium iodid (PI) staining buffer (50 µl/ml PI, RNAse A; Beckman Coulter, Krefeld, Germany) and incubated for 15 min at 37°C. Cell cycle distribution was analysed by FACSCalibur (BD Biosciences, San Diego, CA) using ModFit software.

### Cell Cycle Checkpoint Analysis by Western Blot

For total protein analysis, cells were resuspended in lysis buffer (50 mM Tris-HCl pH 7.4, 150 mM NaCl, 50 mM NaF, 1 mM EDTA, 10% NP-40, 1% Triton-X and protease inhibitors), incubated on ice for 10 min and centrifuged at 15,000 rpm for 15 min. Aliquots of protein extracts (20 µg) were separated on 12% SDS-PAGE and electroblotted onto 0.45 µm Hybond ECL nitrocellulose membranes (Amersham Biosciences, Little Chalfont, UK). The membranes were blocked with 3% milk blocking buffer for 1 h and incubated with the primary antibodies for 2 h at room temperature. Primary antibodies against CDK2, cdc2 (CDK1), cyclin B1, p27^Kip1^, p53, and β-actin were purchased from Santa Cruz (Santa Cruz Biotechnology, Santa Cruz, CA). Blots were developed using horseradish peroxidase-conjugated secondary antibodies (Dako, Vienna, Austria) at room temperature for 1 h and the SuperSignal® West Pico Chemoluminescent Substrate (Thermo Scientific, Rockford, IL), in accordance with the manufacturers’ protocol.

### Human MMP Fluorokine® MAP

STS cells were treated with the respective IC_50_ of costunolide or dehydrocostus lactone for 24 and 48 h. The cell culture supernatant was 5-fold diluted. One hundred microliters of Calibrator Diluent RD5–37 were added and mixed thoroughly. The samples for the Human MMP Base Kit were prepared according to the manufacturer’s instruction (R&D Sytems, Vienna, Austria). Microparticles were detected using the 100 suspension array system (BioPlex 200, Munich, Germany) within 90 min. The reader was set to read a minimum of 100 beads with identical unique detection signal and results were expressed as median fluorescent intensity (MFI).

### Real-time RT-PCR

Real-time RT-PCR was performed according to MIQE criteria [19] to determine the relative expression of MMP-1, -2, and -9 levels. Total RNA was isolated from treated and untreated cells with RNeasy Mini Kit (Qiagen, Hilden, Germany), following the manufacturer’s recommended protocol. RNA quality was analyzed using the Agilent RNA 6000 Nano Kit and the Bioanalyzer 2100 (Agilent Technologies, Santa Clara, CA). RIN values were between 9.8 and 10.0. DNA was digested with 1 U DNase (Fermentas, St.Leon-Rot, Germany) per µg RNA. One µg RNA was reverse transcribed using RevertAid cDNA Synthesis Kit (Fermentas). Real-time PCR reactions were performed in triplicates using the Platinum SYBR Green Super Mix with ROX (Invitrogen) on an AB7900HT (Applied Biosystems, Invitrogen). Table 1 lists the primers used for real-time PCR. CDK2 RT-PCR was performed with the QuantiTect primer assay (Qiagen). The expression levels were normalized (ΔCt) to the expression of the housekeeping genes glyceraldehyde 3-phosphate dehydrogenase (GAPDH), β-actin (ACTB) and hypoxanthine phosphoribosyltransferase (hprt-n) as an internal control and compared to the corresponding ΔCt (ΔΔCt) of control cells. *p<0.05 [20].

**Table 1:** Primer Sequences used for RT-PCR.

target gene	name	primer	oligonucleotide sequence 5′-3′
MMP-1	matrix metalloproteinase 1	forward	CTG TTC AGG GAC AGA ATG TGC T
	(interstitial collagenase)	reverse	TCG ATA TGC TTC ACA GTT CTA GGG
MMP-2	matrix metalloproteinase 2	forward	TGA TCT TGA CCA GAA TAC CAT CGA
	(gelatinase A)	reverse	GGC TTG CGA GGG AAG AAG TT
MMP-9	matrix metalloproteinase 9	forward	GGG ACG CAG ACA TCG TCA TC
	(gelatinase B)	reverse	TCG TCA TCG TCG AAA TGG GC
GAPDH	glyceraldehyde 3-phosphate	forward	AAG GTC GGA GTC AAC GGA
	dehydrogenase	reverse	ACC AGA GTT AAA AGC AGC CCT
hprt-n	hypoxanthine	forward	ATG GGA GGC CAT CAC ATT
	phosphoribosyltransferase	reverse	ATG TAA TCC AGC AGG TCA GCA A
ACTB	β-actin	forward	CTG GAA CGG TGA AGG TGA CA
		reverse	AAG GGA CTT CCT GTA ACA ATG CA

### Invasion Assay

STS cells were pre-treated with 3 and 10 µg/ml of each compound for 24 h. After this incubation period, cells were stained with the Vybrant® DiI Cell-Labeling Solution (Molecular Probes®, Invitrogen) for 1 h at 37°C. The top chamber of 8 µm pore size HTS FluoroBlok™ cell culture inserts (BD Biosciences) were pre-coated with 100 µl 1∶40 Matrigel Basement Membrane Matrix (BD Biosciences) and 5×10^4^ DiI stained cells were seeded per insert in 500 µl serum-free DMEM-F12 medium. The inserts were placed into 24-well-plates containing 800 µl DMEM-F12 with 5% FBS per well. Cells were incubated for 24 h at 37°C. Invasive cells on the lower side of the membrane were counted by fluorescence microscope. Experiments were performed in triplicate and three random fields per membrane were counted for quantification.

### Statistical Analysis

All values are expressed as mean values ± SD. Student’s unpaired *t*-test was used to evaluate differences between treated groups and their respective controls. The significance of dose or time responses was assessed by repeated measures analysis. Graphic data were prepared with SigmaPlot® (Systat Software Inc., San Jose, CA).

## Results

### Costunolide and Dehydrocostus Lactone Reduced Cell Proliferation and Viability of STS Cells

**Figure 1:**
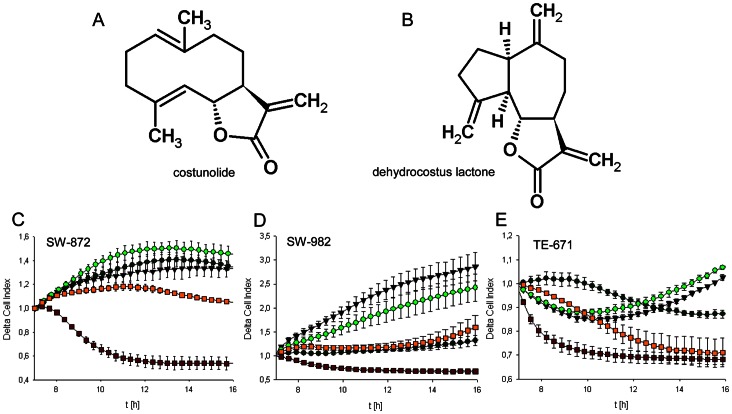
Proliferation analysis of sarcoma cells. Chemical structures of A) costunolide and B) dehydrocostus lactone. STS cells were seeded at 10,000 per well and measured with the xCELLigence system. Dynamic proliferation curves for C) SW-872, D) SW-982, and E) TE-671 sarcoma cells in the presence of 0 (black triangles), 3 µg/ml (dark green circles), and 10 µg/ml costunolide (light green circles), or 3 µg/ml (yellow squares), and 10 µg/ml (red squares) dehydrocostus lactone. Both substances inhibited cell growth in a concentration dependent manner.

The chemical structures from costunolide and dehydrocostus lactone isolated from *Saussurea lappa* were represented in Figure 1A and 1B. STS cells were treated with 3 and 10 µg/ml costunolide and dehydrocostus lactone for 48 h. During this period, cell growth curves were automatically recorded in real time by the xCELLigence System. The most notable changes were observed in the first 16 h (Fig. 1). Both substances inhibited cell growth in a concentration dependent manner in all three cell lines tested. For SW-872 cells, dehydrocostus lactone already blocked cell growth at a concentration of 3 µg/ml (Fig. 1C). The concentration of 10 µg/ml caused a complete reduction of cell proliferation. SW-982 and TE-671 cells demonstrated a high sensitivity for dehydrocostus lactone illustrated by a clear lowered cell index at a concentration of 3 and 10 µg/ml (Fig. 1D–E). A block of cell growth or rather an induced cell death may contribute to this decrease in cell index. Subsequent to the continuous xCELLigence cell monitoring, the slope representing the rate of change of the cell index was calculated from time 7–24 h and presented in Table 2. Data shown are representative from three independent experiments.

**Table 2:** Slope values calculated from xCELLigence real-time measurements between 7–24 h.

	SLOPE (1/h)					
	*costunolide*			*dehydrocostus lactone*		*control*
	3 µg/ml	10 µg/ml	20 µg/ml	3 µg/ml	10 µg/ml	0 µg/ml
**SW-872**	0.002±0.003	0.004±0.002	−0.040±0.002	−0.005±0.001	−0.023±0.003	0.028±0.001
**SW-982**	0.111±0.003	0.079±0.004	−0.016±0.001	0.173±0.009	−0.008±0.002	0.116±0.002
**TE-671**	0.031±0.001	0.001±0.002	−0.077±0.004	−0.012±0.002	−0.007±0.001	0.025±0.001

### Dehydrocostus Lactone Caused an Increase of S and G2/M Phase Cells

**Figure 2:**
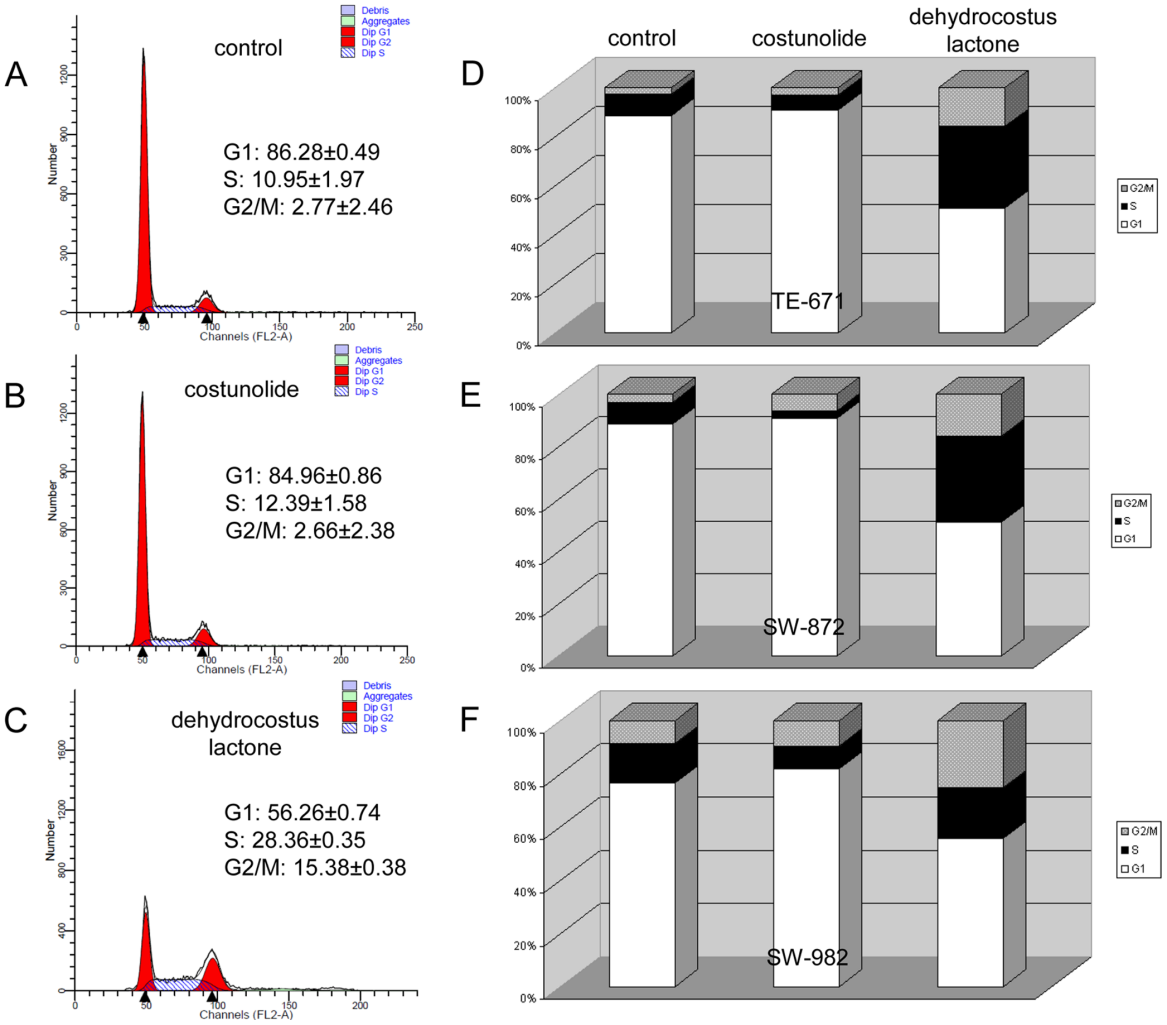
Influence of sesquiterpene lactones on cell cycle distribution. A) Untreated TE-671 cells were measured as control. B) Treatment of TE-671 cells with the IC_50_ of costunolide led to a very small change in the distribution, whereas, C) the IC_50_ of dehydrocostus lactone caused a significant decrease in the number of cells in G1 phase after 48 h which was accompanied by an increase of the number of S and G2/M phase cells. Values are expressed as percentage of the cell population in the G1, S, and G2/M phase of cell cycle. Graphical representations of the cell cycle distribution of D) TE-671, E) SW-872, and F) SW-982 cells.

To investigate the effects of costunolide and dehydrocostus lactone on cell cycle, STS cells were exposed to their respective IC_50,_ which was determined by our group in a previous study [9]. Representative FACS measurements from the rhabdomyosarcoma TE-671 cell line are shown in figure 2A–C. Untreated cells were measured as control (Fig. 2A). Interestingly, when treated with costunolide, no significant changes in cell cycle distribution were detected compared to untreated control groups (Fig. 2B). In contrast, dehydrocostus lactone (Fig. 2C) caused a decrease in the number of cells in G1 phase (56.26±0.74% of the cell population compared with 86.28±0.49% for the controls) which was accompanied by an increase of the number of S (28.36±0.35% of the cell population compared with 10.95±1.97% for the controls) and G2/M phase cells (15.38±0.38% of the cell population compared with 2.77±2.46% for the controls). These results were also evident for the two other cell lines. After dehydrocostus lactone treatment the liposarcoma cell line SW-872 showed very similar values (G1 phase: 50.81±0.83% vs 88.60±0.24% for the controls; S phase: (33.25±0.95% vs 8.51±0.27% for the controls; and G2/M phase: 15.95±1.77% vs 8.89±0.50% for the controls). However, the synovial sarcoma SW-982 cells showed the shift of the cell cycle distribution to a lesser extent (G1 phase: 55.71±1.70% vs 76.63±0.33% for the controls; S phase: (19.13±0.15% vs 14.83±0.45% for the controls; and G2/M phase: 25.17±1.84% vs 8.54±0.12% for the controls). The graphical representations of the cell cycle distribution are summarized for TE-671 (Fig. 2D), SW-872 (Fig. 2E), and SW-982 (Fig. 2F).

### Dehydrocostus Lactone Decreased cdc2, Cyclin B1 and CDK2 Levels

**Figure 3:**
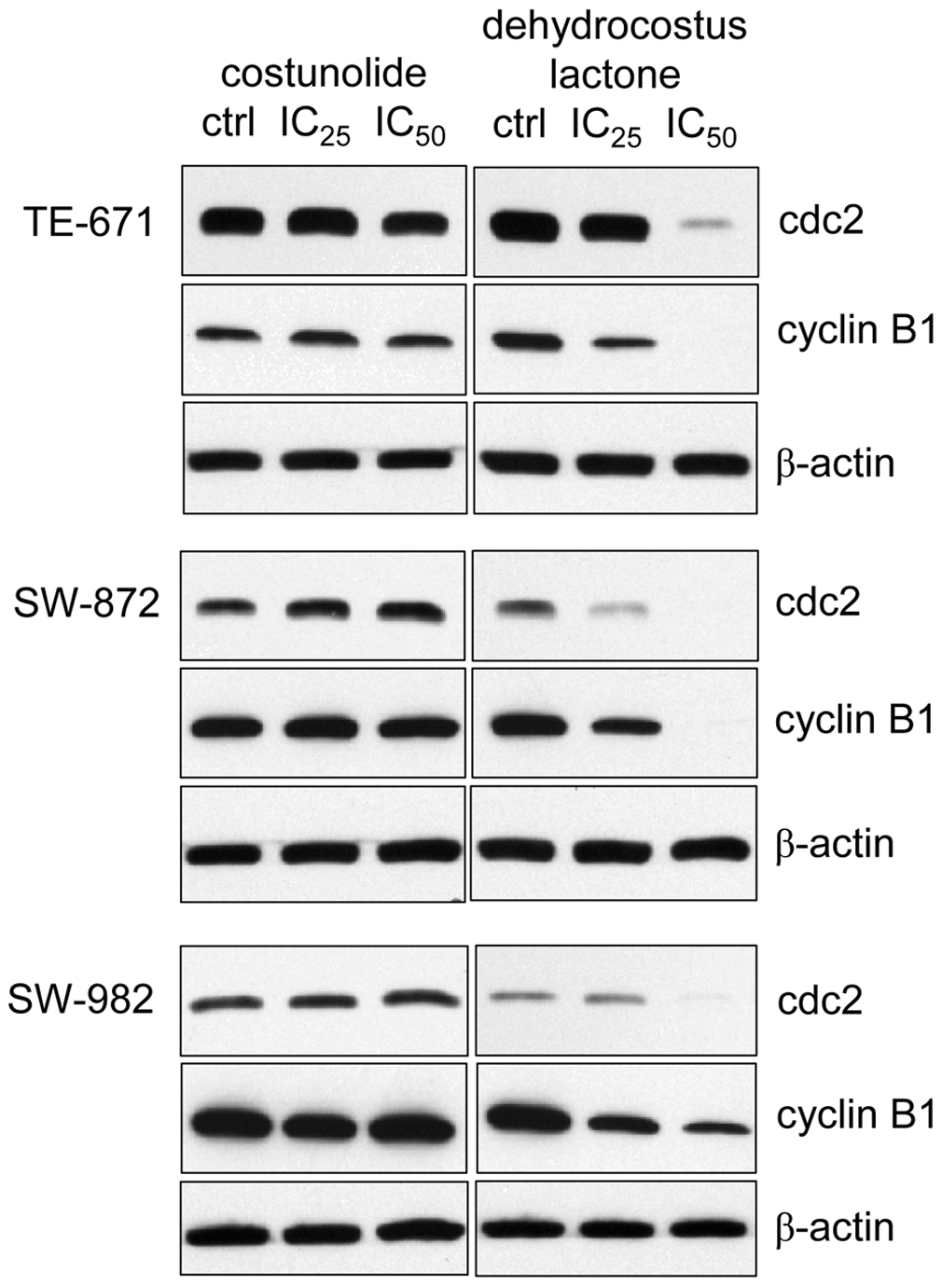
Western blot analysis of G2/M arrest regulator proteins. Total protein analysis revealed a significant downregulation of cdc2 and cyclin B1 in dehydrocostus lactone treated STS cells, whereas, costunolide treatment did not affect the protein levels of these G2/M checkpoint regulators. β-actin was used as loading control. Data shown are representative from at least three independent experiments.

**Figure 4:**
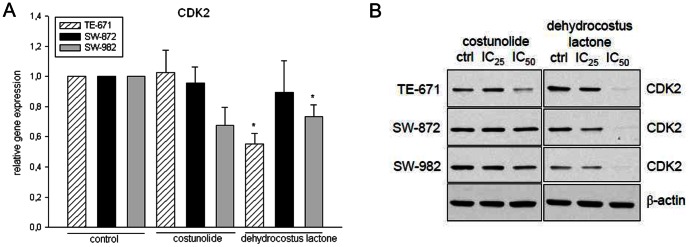
Relative mRNA expression and Western blot analysis of CDK2. A) Dehydrocostus lactone decreased the CKD2 mRNA expression levels significantly in SW-982 (*p = 0.041) and TE-671 (*p = 0.007) cells. B) This downregulation was also found on the protein level. No significant alteration could be observed after costunolide treatment. β-actin was used as loading control. Data shown are representative from at least three independent experiments.

**Figure 5:**
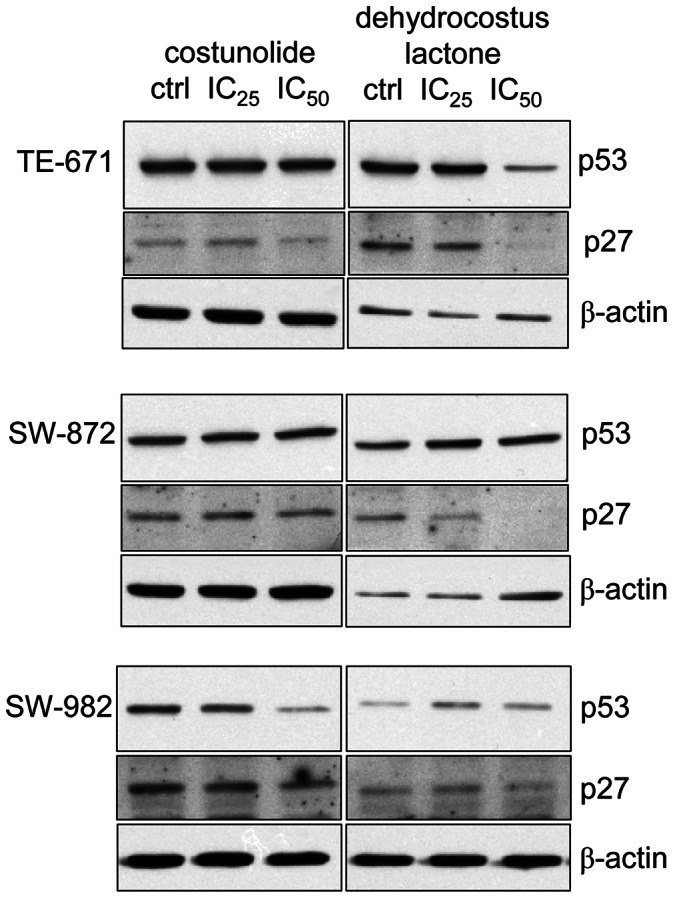
Western blot analysis of p53 and the CDK inhibitor p27^Kip1^ proteins. Total protein analysis confirmed no significant changes in the p53 expression after costunolide or dehydrocostus lactone treatment. In dehydrocostus lactone treated TE-671 and SW-872 cells, the expression of the CDK inhibitor p27^Kip1^ was reduced. β-actin was used as loading control. Data shown are representative from at least three independent experiments.

Furthermore, we examined the specific regulatory proteins responsible for cell cycle arrest under the exposure of IC_25_ and IC_50_ of each sesquiterpene lactones for 48 h. Compared to control cells, the expression of cdc2 and cyclin B1 was clearly declined in dehydrocostus lactone-treated STS cells. Corresponding to the FACS data, costunolide did not affect the expression of these G2/M checkpoint regulator proteins (Fig. 3). Reverse transcriptase PCR analysis revealed a substantial decrease in CDK2 mRNA levels in all three cell lines under dehydrocostus lactone treatment, whereas costunolide showed no effect (Fig. 4A). Correspondingly, immunoblotting of STS cell lines showed diminished CDK2 levels following 48 h treatment with dehydrocostus lactone in dose dependent manner (Fig. 4B). Previous findings demonstrated that p53 activation and induction of the CDK inhibitors p21^Cip1^ and p27^Kip1^ are involved in G1- and G2 arrest as well as in response to DNA damage. Therefore, we investigated whether p53 and p27^Kip1^ might be induced by sesquiterpene lactones treatment. Western Blot analysis revealed the expression of p53 in all groups. Assaying STS for the expression of the CDK inhibitor p27^Kip1^ resulted in a clear removal of p27^Kip1^ expression under dehydrocostus lactone in TE-671 and SW-872 cells (Fig. 5).

### Costunolide and Dehydrocostus Lactone Affected MMP Levels and Invasion Potential of STS Cells

**Figure 6:**
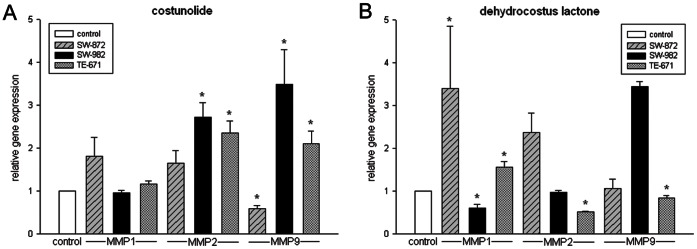
Relative mRNA expression of MMP-1, -2 and -9 measured by real-time RT-PCR. A) Costunolide increased MMP-2 and -9 expression levels significantly in SW-982 and TE-671 cell lines. B) Dehydrocostus lactone increased MMP-1 expression level significantly in SW-872 and TE-671 cells. Furthermore, the levels of MMP-2 and -9 were significantly reduced in TE-671, but increased in SW-982 cells.

**Figure 7:**
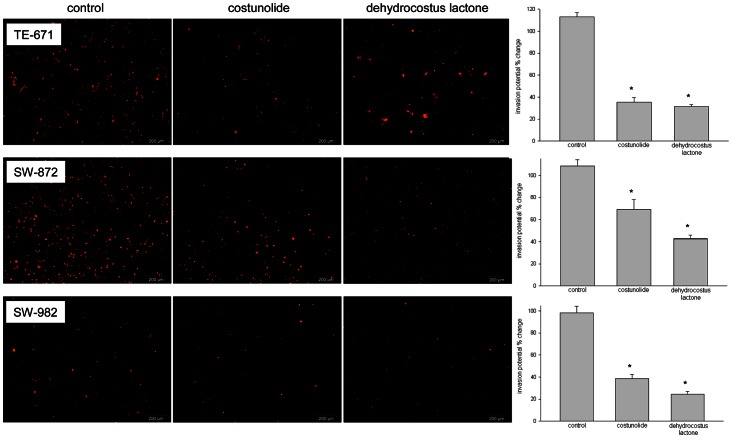
Sesquiterpene lactones inhibited the invasion potential. Costunolide and dehydrocostus lactone pre-treated DiI stained STS cells were seeded in the upper compartment of matrigel coated 8 µm pore size HTS FluoroBlok™ cell culture inserts in serum free medium. Invasion towards the lower compartment containing medium supplemented with 5% FBS was monitored by fluorescent microscope and quantified after overnight incubation at 37°C. The invasion potential was significantly reduced in all three STS cell lines.

To investigate whether the compounds affect the expression of MMP-1, -2, and -9, the human MMP Fluorokine® MAP assay and RT-RCR experiments were performed. Cells were treated with their respective IC_50_. As shown in Table 3, MMP-1 levels represented by mean fluorescence intensity (MFI) were significantly upregulated in dehydrocostus lactone-treated SW-872 cells. Costunolide treatment did not significantly change the MFI for MMP-1 in one of the three cell lines. On the other hand, MMP-2 and -9 levels were significantly increased in costunolide treated SW-982 and TE-671 cells. However, treatment with dehydrocostus lactone significantly reduced these expressions in TE-671 cells, but increased the MMP-9 level in SW-982 cells. Furthermore, we were able to confirm our observations by real-time RT-PCR experiments in revealing a significant increase in MMP-1, -2, and -9 mRNA levels in SW-872 cells under dehydrocostus lactone treatment (Fig. 6). In addition, sesquiterpene lactones treatment resulted in a decreased invasion potential of STS cells. SW-872 cells were significantly reduced from 108.00±15.12% (control) to 69.43±19.26% (p = 0.0069) for costunolide-treated cells and 42.59±8.25% (p = 2.52E-6) for dehydrocostus lactone-treated cells. A similar change was observed in SW-982 cells (98.06±13.85% vs 38.71±10.03% (p = 8.52E-5) vs 24.49±6.18% (p = 8.56E-05)) and TE-671 cells (112.91±8.06% vs 35.35±8.65% (p = 1.26E-05) vs 31.53±3.62% (p = 3.80E-05)) (Fig. 7).

**Table 3:** MMP-1, -2, and -9 expression measured with the Luminex® system.

	MMP-1 expression [MFI]			MMP-2 expression [MFI]			MMP-9 expression [MFI]		
time	*control*	*costunolide*	*dehydrocostus*	*control*	*costunolide*	*dehydrocostus*	*control*	*costunolide*	*dehydrocostus*
[h]			*lactone*			*lactone*			*lactone*
**SW-872**									
*24*	10749±412	7929±107	**18823±1374**	2396±290	1190±207	**1264±63**	229±9	**147±22**	399±26
*48*	23809±151	22812±350	23901±113	7215±727	3842±260	**1264±63**	507±35	**253±13**	609±35
**SW-982**									
*24*	24420±166	21375±962	26227±524	251±36	**521±98**	**134±10**	27±1	**47±5**	42±6
*48*	26972±603	25086±571	27416±578	678±60	920±60	**141±132**	26±7	**52±4**	**69±6**
**TE-671**									
*24*	34±4	39±10	40±5	160±10	**304±118**	**47±9**	41±4	69±32	**6±2**
*48*	83±14	74±8	86±4	746±75	879±94	**263±20**	61±5	78±7	**6±2**

STS cells were treated with the IC_50_ concentrations of costunolide or dehydrocostus lactone for 24 and 48 h. Results are represented by mean fluorescence intensity (MFI) and p-values. Bold numbers represent significant differences between control and treated cells (p>0.05).

## Discussion

Costunolide and dehydrocostus lactone have been reported as major and biologically active compounds of the roots of *Saussurea lappa* (Asteraceae), a well-known Asian traditional herbal medicine [1]–[3]; [5]–[7]; [21]–[23]. The pro-apoptotic effect of these compounds in different human cancer cells has also been reported [9], [24], [25]. In the present study, we examined the effect of these compounds on the expression of cell cycle checkpoint proteins in STS cell lines. The results of fluorescence-activated cell sorting of STS cells treated with their IC_50_ values of dehydrocostus lactone showed a significant cell cycle arrest at the G2/M interface, suggesting this as a mechanism for the antiproliferative effect of this sesquiterpene lactone. The cell cycle is tightly regulated through a complex network of positive and negative cell-cycle regulatory molecules such as cyclin-dependent kinases (CDKs), CDK inhibitors, and cyclins. Cell cycle checkpoints are available to allow cellular repair, and in most cases, checkpoints may result in the activation of apoptosis signalling, if the cellular damages are too serious to be repaired properly. The CDKs are a family of enzymes pivotal in the progression of the cell cycle, their activity being facilitated by partnership with cyclins.

The CDK activities are regulated by cell cycle inhibitors of the cip/kip family, e.g. p21 or p27, which has been shown to arrest cells in the G1 or G2 phase of the cell cycle [26]. We observed in the cell cycle analysis that dehydrocostus lactone-treatment resulted in a significant decrease in the number of cells in G1 phase after 24 h which was accompanied by an increase of the number of S and G2/M phase cells, via the down-regulation of cdc2 (CDK1) together with cyclin B1. In contrast, costunolide treatment demonstrated no changes in the regulation of cdc2 as well as cyclin B1, which matches the observed unaltered cell cycle distribution. Several investigators have shown that cdc2 in combination with cyclin B1 is critical in the G2/M phase transition [27], [28]. The altered pattern of cell cycle-related regulator protein expression induced by dehydrocostus lactone treatment is consistent with an arrest at G2/M because these proteins are not expressed in resting cells. Thus, G2/M arrest via cdc2 down-regulation may be an important molecular mechanism by which dehydrocostus lactone inhibits cancer cell growth. Furthermore, our results demonstrated that CDK2 levels decreased in STS cell lines under treatment with dehydrocostus lactone. Equally, the measurement of CDK2 mRNA levels displayed a significant reduction in the expression following treatment with dehydrocostus lactone for 24 h.

The tumor suppressor gene p53 is regarded as a key factor in the balance between cell survival and cell death via regulation of both the G1 and G2/M phases of the cell cycle [29]. Previously, it was reported that p53 induced cell cycle regulators including p21 [30], which in turn initially inhibits the cdc2-cylinB1 complex and subsequently reduces cyclin B1 and cdc2 protein levels, leading to G2-arrest [31]. Treatment of STS cell lines expressing wild type p53 with both compounds caused no significant changes. However, dehydrocostus lactone caused a clear decrease of p27^Kip1^ expression.

Our second question of interest was whether costunolide or dehydrocostus lactone influence the expression of MMPs and reduce the invasion potential of STS cell lines. SW-982 cells have been reported to highly express MMP-1 (collagenase-1 or interstitial collagenase), while MMP-2 (Gelatinase A) was only weakly expressed and for MMP-9 (Gelatinase B) no gene expression has been detected [32]. Pazzaglia et al. demonstrated that MMP-2 in general was highly produced in SW-872 cells, while MMP-9 detection was minimal [33]. In TE-671 cells, MMP expression was reported as low abundant [34]. Using the human MMP Fluorokine® MAP assay and real-time RT-RCR experiments, we detected measureable amounts of MMP-1, -2, and -9 in all three cell lines. The TE-671 cell line exhibited the lowest level of MMPs, while SW-872 cells displayed the highest quantity of MMPs in the Fluorokine® MAP assay. Cell’s exposure to costunolide or dehydrocostus lactone evoked significant changes in expression of MMPs. Manello et al. showed that MMP-1 is involved in breast cancer initiation/progression, in the metastatic process and could play an initial role in diagnostic, prognostic and therapeutic potential [35]. MMP-1 expression in chondrosarcoma cells correlates with cellular invasion and a transient down regulation of MMP-1 expression decreases cell invasion *in vitro* [36]–[38]. Our results indicate that MMP-1 expression tends to be up-regulated in the presence of costunolide or dehydrocostus lactone, while only in dehydrocostus-lactone-treated SW-982 cells it was significantly down-regulated. MMP-2 and -9 are soluble MMPs which degrade gelatine and play key roles in tumor angiogenesis, growth and metastasis [11]. Several studies have demonstrated their role in sarcoma progression [7], [39]–[42]. Our results have shown that MMP-2 and -9 were significantly increased in costunolide-treated SW-982 and TE-671 and dehydrocostus-lactone-treated TE-671 cells. However, dehydrocostus lactone treatment significantly reduced MMP-2 and -9 levels in TE-671 cells, but increased MMP-9 expression in SW-982 cells. Apart from that, exposure to costunolide lowered MMP-9 level in SW-872 cells. Since the activity of MMPs is connected with metastasis, we investigated whether the potential to migrate was reduced in the presence of these compounds. Using the HTS FluoroBlock™ insert system, we could show that the invasion potential after the treatment with both sesquiterpene lactones was significantly reduced.

In summary, although the mechanisms of its anticancer activity were investigated only in an *in vitro* cell system, especially dehydrocostus lactone has shown potent anticancer activity and is an excellent candidate for further *in vivo* studies.
