# Correction: Sesquiterpene lactones downregulate G2/M cell cycle regulator proteins and affect the invasive potential of human soft tissue sarcoma cells

**DOI:** 10.1371/journal.pone.0342821

**Published:** 2026-02-17

**Authors:** Birgit Lohberger, Beate Rinner, Nicole Stuendl, Heike Kaltenegger, Bibiane Steinecker-Frohnwieser, Eva Bernhart, Ehsan Bonyadi Rad, Annelie Martina Weinberg, Andreas Leithner, Rudolf Bauer, Nadine Kretschmer

After publication of this article [1], concerns were raised regarding Figs 4-5. Specifically:

- The Fig 4B dehydrocostus lactone β-actin panel appears similar to the Fig 5 TE-671 costunolide β-actin panel.
- The Fig 4B costunolide β-actin panel appears similar to the Fig 5 SW-872 costunolide β-actin

The corresponding author stated that the β-actin blot for dehydrocostus lactone shown in Fig 4B of [1] is incorrect, whereas the β-actin blot for costunolide in Fig 4B and all panels in Fig 5 of [1] are correct. A revised version of Fig 4 is provided here, in which the appropriate β-actin blots are shown for each individual sarcoma cell line. The original, uncropped blots underlying the updated Fig 4 and Fig 5 in [1] are provided here in S1 File, and the individual-level quantitative data underlying Fig 4A are provided in S2 File.

The corresponding author further stated that the β-actin panels are intentionally duplicated between Fig 5 in [1] and the updated Fig 4, as they are derived from the same membrane, which was cut into strips prior to antibody incubation and subsequently probed with the respective antibodies for analysis.

**Fig 4:**
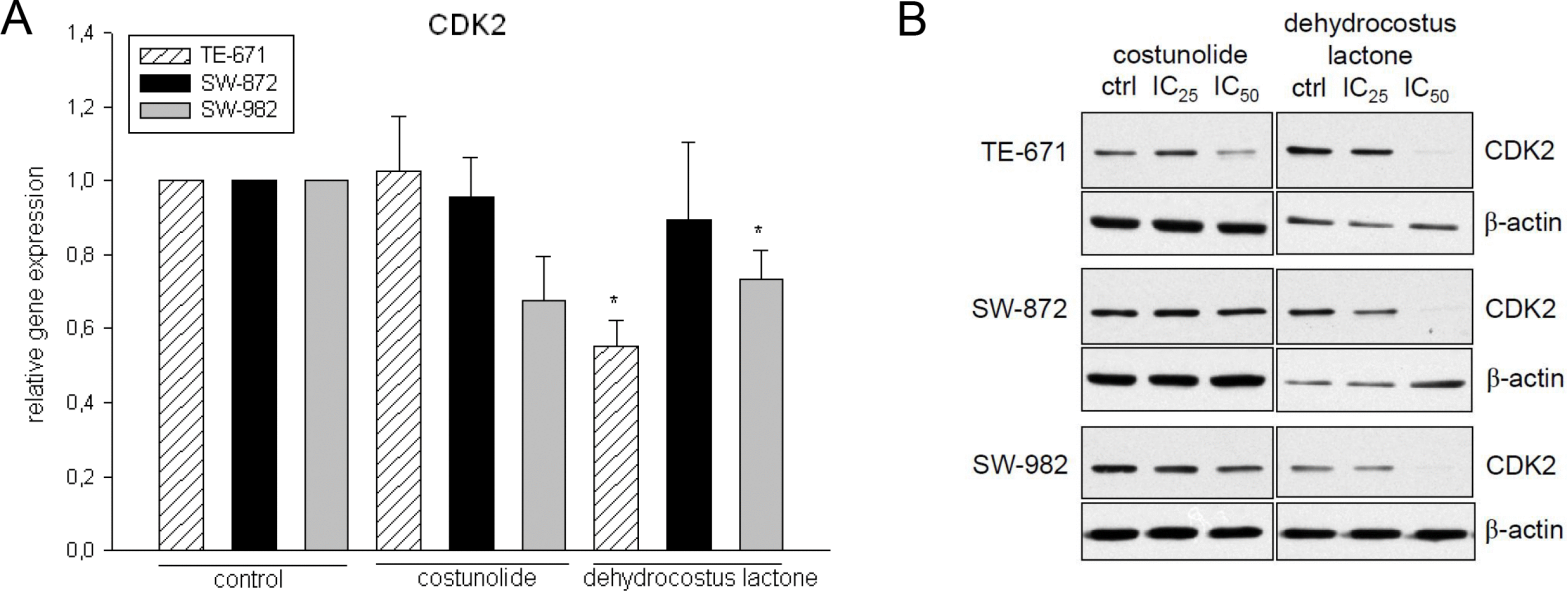
Relative mRNA expression and Western blot analysis of CDK2. **A)** Dehydrocostus lactone decreased the CKD2 mRNA expression levels significantly in SW-982 (*p = 0.041) and TE-671 (*p = 0.007) cells. **B)** This downregulation was also found on the protein level. No significant alteration could be observed after costunolide treatment. b-actin was used as loading control. Data shown are representative from at least three independent experiments.

## Supporting information

S1 File Original uncropped blots in support of Figures 4 and 5. (PDF)

S2 File Individual-level underlying quantitative data for Figure 4A. (XLS)

## References

[1] Lohberger B, Rinner B, Stuendl N, Kaltenegger H, Steinecker-Frohnwieser B, Bernhart E, et al. Sesquiterpene lactones downregulate G2/M cell cycle regulator proteins and affect the invasive potential of human soft tissue sarcoma cells. PLoS One. 2013;8(6):e66300. doi: 10.1371/journal.pone.0066300 23799090 PMC3682952

