# Supplementary material for: Correction: Sesquiterpene lactones downregulate G2/M cell cycle regulator proteins and affect the invasive potential of human soft tissue sarcoma cells
Source: PLoS One. 2026 Feb 17;21(2):e0342821. doi: 10.1371/journal.pone.0342821 (PMC12912533; doi:10.1371/journal.pone.0342821)
Supplement: S1 File — (PDF) [file pone.0342821.s001.pdf]

Costunolide = SLP1
Dehydrocostus lactone = SLP2

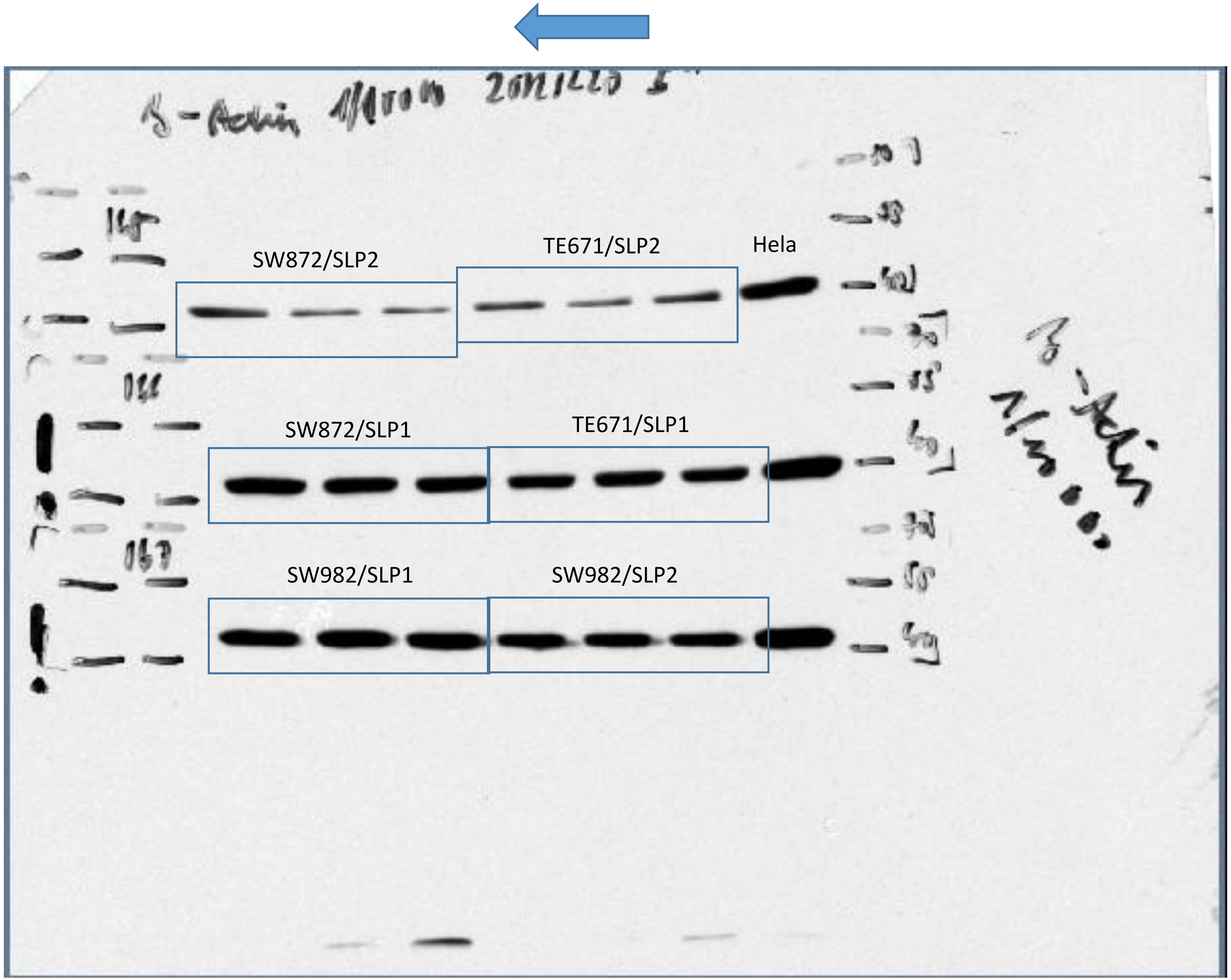

HeLa P54 WB NEU
te671 20121206 24h NK
te671 20121206 24h SLP2 IC25
te671 20121206 24h SLP2 IC50
sw872 24h NK WB
sw872 24h SL-P2 IC25 WB
sw872 24h SL-P2 IC50 WB

HeLa P54 WB NEU
te671 20121207 48h NK
te671 20121207 48h SLP1 IC25
te671 20121207 48h SLP1 IC50
sw872 48h NK WB
sw872 48h SL-P1 IC25 WB
sw872 48h SL-P1 IC50 WB

HeLa P54 WB NEU
sw982 24h NK WB
sw982 20121206 24h SLP2 IC25
sw982 20121206 24h SLP2 IC50
sw982 48h NK WB
sw982 48h SL-P1 IC25 WB
sw982 48h SL-P1 IC50 WB

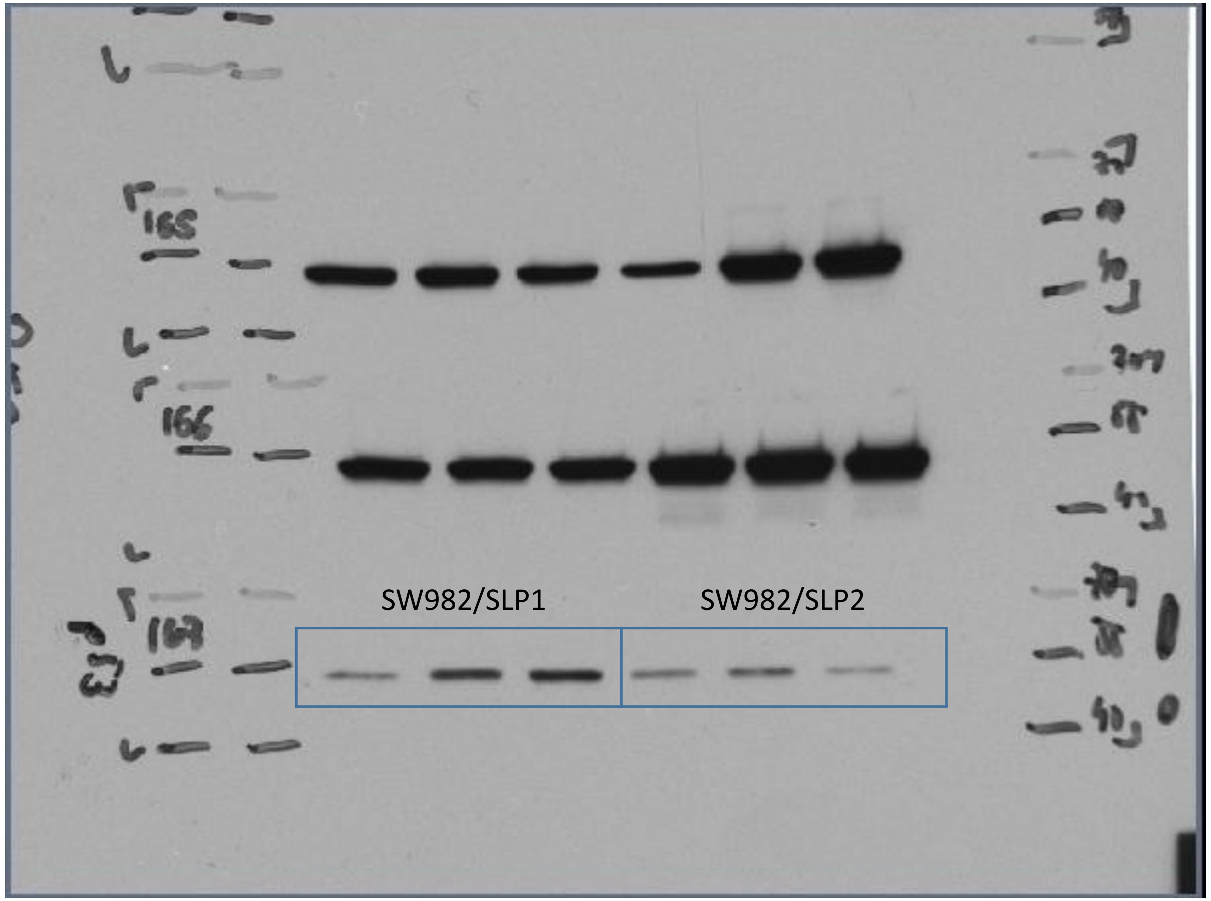
SW982/SLP1
SW982/SLP2

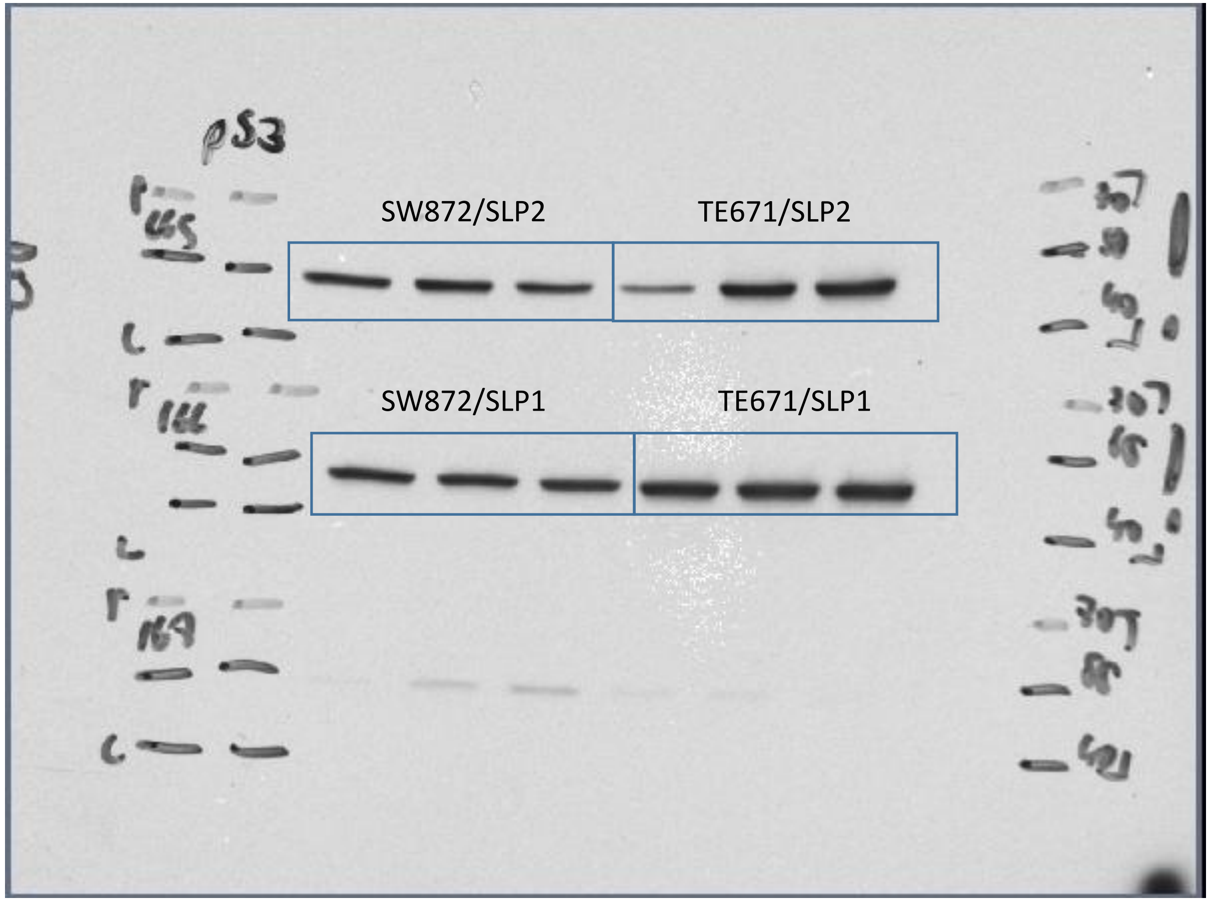
p53
SW872/SLP2
TE671/SLP2
SW872/SLP1
TE671/SLP1

p53

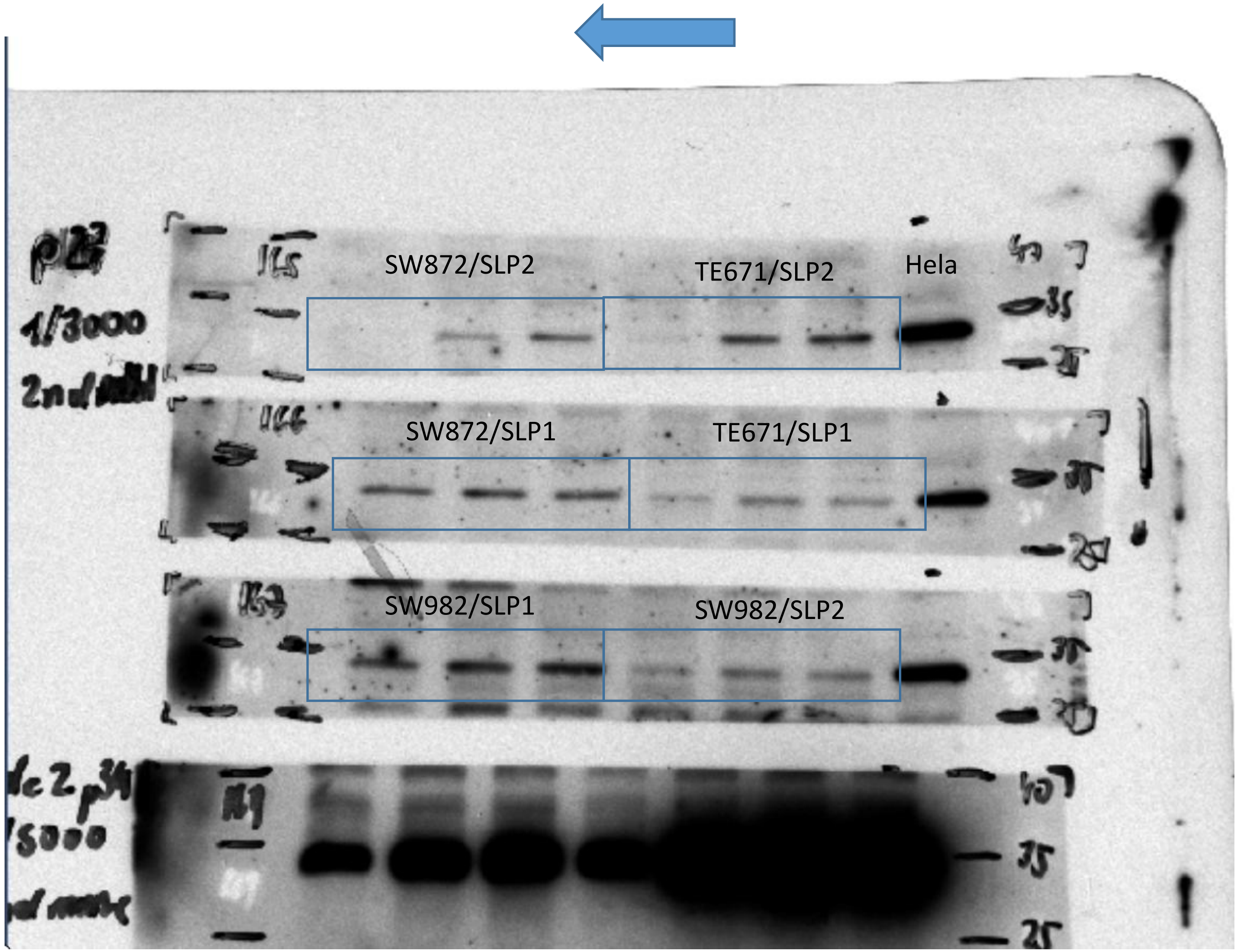
SW872/SLP2
TE671/SLP2
Hela
SW872/SLP1
TE671/SLP1
SW982/SLP1
SW982/SLP2

p27

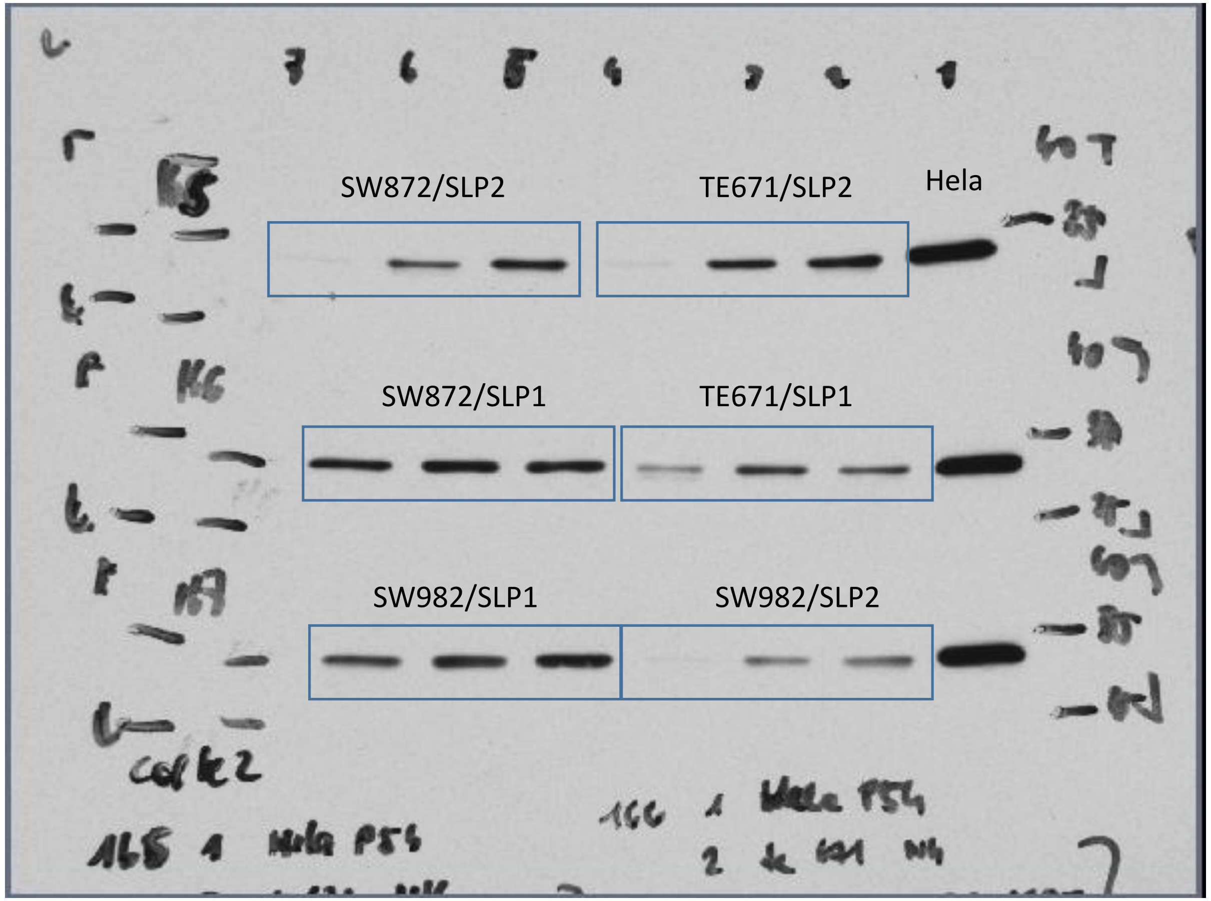
SW872/SLP2
TE671/SLP2
Hela
SW872/SLP1
TE671/SLP1
SW982/SLP1
SW982/SLP2

CDK2
